# Supplementary material for: Sub-resolution contrast in neutral helium microscopy through facet scattering for quantitative imaging of nanoscale topographies on macroscopic surfaces
Source: Nat Commun. 2023 Feb 17;14:904. doi: 10.1038/s41467-023-36578-x (PMC9938237; doi:10.1038/s41467-023-36578-x)
Supplement: Supplementary file 1 — Supplementary Information File [file 41467_2023_36578_MOESM1_ESM.pdf]

Supplementary Information

Sub-resolution contrast in neutral helium microscopy through facet scattering for quantitative imaging of nanoscale topographies on macroscopic surface

Sabrina D. Eder†[1], Adam Fahy†[2], Matthew G. Barr†[2], J. R. Manson[3], Bodil Holst[1], Paul C. Dastoor[2]

Supplementary Discussion 1: SHeM contrast

Supplementary Note 1: SHeM contrast modelling rationale

In the Methods section we described the underlying process by which relative scanning helium microscope (SHeM) intensities (and hence contrast) is modelled. This modelling can be summarised in the following manner: For each sample a height map is obtained (either directly using an atomic force microscope (AFM), or estimated from scanning electron microscope (SEM) micrographs); the SHeM geometry is imported; atoms are ray-traced from a model SHeM source to the height map where they are reflected assuming a specular scattering process; finally the scattered atoms are traced further to determine the relative fraction that strike the detector aperture.

In this Supplementary Discussion we provide a rationale as to why this modelling is reasonable. We base our argument on established helium atom scattering (HAS) theory, alongside energy-resolved helium atom scattering experiments (time-of-flight, TOF) performed on samples analogous to those measured in the SHeM. Further support is also found in an additional set of SHeM micrograph studies, where the helium beam temperature (and hence mean beam energy) is varied. See the Methods section for a brief description of how these experiments were performed.

Supplementary Note 2: Helium atom scattering - an introduction

The interaction between an incident helium atom beam and a surface has been extensively studied experimentally and theoretically [1, 2, 3]. To justify our model, we start out by briefly summarising the possible scattering processes that can occur; we then present a set of experiments that support our choice of model.

In order to justify our model, the first major distinction we need to make is the differences between elastic and inelastic scattering. In the case of elastic scattering, the energy of the helium atom is unchanged during the scattering process. In the case of inelastic scattering, there is an energy exchange with the surface and the energy of the reflected helium atom is decreased or increased through phonon creation or annihilation on the surface.

Thermal helium atoms have a wavelength of the same order as atomic spacings in materials, so scattering can occur in the form of diffraction if the substrate is crystalline with a corrugated surface electron density distribution and reciprocal lattice parameters matching the **k**-vector component of the helium atom parallel to the surface. Elastic scattering can also occur as resonant state scattering, also referred to as selective adsorption resonance. These resonances occur if the **k**-vector component of the helium atom perpendicular to the surface matches a trapped state in the helium-surface interaction potential well. It is observed on atomically flat substrates as a dip or peak in reflection intensity in a particular direction, as the atoms are now moving along the surface until they exit the bound state via a second diffraction, or are scattered from defects at a random angle. Finally, so called diffuse elastic scattering can occur, which happens in the case of a rough surface, whereby the atoms scatter elastically in many different directions. Note that any observed change in recorded time-of-flight here reflects a change in path length due to the changed scattering angle, rather than a change in energy.

For inelastic scattering, we must distinguish between the single phonon and multi phonon regimes. In the single phonon regime, the helium atoms excite or de-excite individual vibration modes. The single phonon regime occurs when the energy of the incident helium atoms "matches" discrete excitation energies of surface charge oscillations (the single phonons). Phonon modes defined completely by the parallel component of their wavevector give rise to distinct peaks, for example Rayleigh modes, ZA modes, etc. The single-phonon contribution arising from a single-quantum transfer of bulk phonons give rise to a single-phonon continuum. The phonon energy in the single phonon regime provides chemical information about the surface composition, because different substrates and adsorbate molecules have distinct phonon signatures.

In the multi-phonon regime several phonons are excited at the same time. This situation occurs if several different adsorbates are present and/or the vibration energies for the surface molecule charge oscillations are much lower than the energy of the incident helium atoms (the helium atoms see the surface molecules as "floppy"). In this case there will not be discrete excitations, but rather a broad, continuous TOF spectrum. The multi-phonon regime is also referred to as diffuse inelastic scattering.

Inelastic and elastic scattering can occur simultaneously. In a TOF spectrum the elastic contribution appears at the point of zero energy transfer. The most extreme case of inelastic scattering, Knudsen flux scattering, can be identified from the most probable velocity measured (see explanation below).

From the discussion above we can summarise that our model is assuming that the contrast comes from diffuse elastic scattering. We do not include phenomena such as diffraction or resonant state scattering or multiple scattering. Two recent publications on theoretical methods for calculating resolutions in a SHeM take a different approach to contrast than our model and assume a $cos(\theta)$ distribution of the scattered lobe with respect to the local surface normal of the sample [4, 5]. In other words, the scattered lobe is independent of the angle of the incident beam with respect to the local surface normal. In light optics the $cos(\theta)$ distribution is equivalent to Lambertian reflection, which is also the term referred to in the papers [4, 5]. Note that this type of scattering is different from that used in the model, where the scattering is dependent on the angle between the beam and the local surface normal. The $cos(\theta)$ distribution is also used to model experimental data in other papers, see for example [6]. Here the scattering is referred to as diffuse scattering. We point out that the $cos(\theta)$ scattering distribution is also the angular dependence

[1]Department of Physics and Technology, University of Bergen, Bergen, Norway. [2]Centre for Organic Electronics, University of Newcastle, Callaghan, NSW 2308, Australia. [3]Department of Physics and Astronomy, Clemson University, South Carolina 29634, U.S.A. †These authors contributed equally to this work. E-mail: paul.dastoor@newcastle.edu.au.

that arises in Knudsen flux scattering [7] as also mentioned in [8]. However, there is a fundamental difference between Lambertian reflection and Knudsen flux scattering. In Lambertian reflection the light does not change its wavelength (energy). This is not the case for the Knudsen flux scattering; the conditions for obtaining what we refer to as Knudsen flux scattering are well known. The Knudsen flux is the flux of particles that would pass in one direction through an imaginary flat plane placed in an equilibrium gas. In the case of molecule-surface collisions, it is the scattered (desorbed) distribution, when the incident beam is totally adsorbed on the surface into the physisorption well, and then remains in the well long enough to equilibrate to the surface temperature, and then ultimately leaves the surface via desorption [9]. We are thus in the fortunate situation that we can easily determine if we have Knudsen flux scattering or not - a helium beam created by a free jet expansion has a narrow velocity distribution with the most probable velocity, v_p, given as [10]:

$$v_p = \sqrt{\frac{5\, k_b T_0}{m_{He}}}, \tag{1}$$

where k_b is Boltzmann's constant, T_0 the beam temperature and m_{He} the mass of the helium atoms. A Knudsen flux distribution scattered beam will have a most probable velocity given as [11]:

$$v_{pM} = \sqrt{\frac{2\, k_b T_S}{m_{He}}}, \tag{2}$$

where T_S is the temperature of the surface. We see that there will be a shift in the most probable velocity even if both beam and surface are at room temperature. This velocity shift can easily be measured using TOF.

Supplementary Note 3: Helium atom scattering experiments

We now move on to present our HAS measurements for the different samples and explain how they justify our diffuse elastic scattering model. Fig. 5 explains the HAS geometrical conditions under which the measurements were performed.

As discussed in the main paper, experiments were carried out on three sample systems: i) Glass of various roughness ii) diamonds of various roughness (nano, intermediate and micro) iii) gold on doped silicon with varying gold layer thickness. The first two systems were deliberately chosen as representing different topographies, while still being chemically similar.

The HAS experiments average over much larger sample areas than does SHeM (mm^2 versus μm^2); the large sampling area is necessary to obtain enough signal for the TOF measurements. The first step for all three systems was to test if the Michelson contrast measured with HAS was similar to the Michelson contrast measured with SHeM. This test was carried out using so called rocking curve measurements, where the total reflected intensity was measured over a range of incident He-beam angles θ_i with respect to the sample surface normal. Note that for the MAGIE setup, the specific nomenclature is that $\theta_i = \alpha_M$ (see also the Methods section). All rocking curves were recorded with the chopper disc removed from the beam line to maximise the recorded signal. These initial measurements were then followed by TOF measurements recorded with the experimental setup either in specular ($\alpha_M = \alpha_D/2$) or off-specular ($\alpha_M \neq \alpha_D/2$). Off-specular can be achieved by either changing the sample rotation angle to $\alpha_M \neq 45°$ or by varying the detector angle to $\alpha_D \neq 90°$. All TOF measurement spectra were then transformed into energy-resolved spectra relative to the initial energy of the helium atoms in the beam.

Supplementary Note 3.1: Glass measurements

Supplementary Fig. 1a shows rocking curve measurements for the three different glass samples. There are no signs of diffraction or resonance scattering over the whole range. Below in Supplementary Fig. 1b the Michelson contrast from HAS is plotted together with the Michelson contrast obtained in SHeM. As can be seen there is a very good agreement between the two.

Supplementary Fig. 1c shows the energy-resolved TOF spectra for a room temperature beam, for a range of incident scattering conditions. The most probable energy in the case of Knudsen flux scattering is indicated as a blue line in all plots. As can be seen in the spectra, there is no indication of Knudsen Flux scattering, with the TOF spectra showing a broad distribution centred around the specular energy. Remember that for diffuse elastic scattering a broad spectrum will be seen due to the different pathlengths the atoms travel. The only clear feature is a slight difference in the overall intensity between the different samples with the elastic scattering signal (at $\Delta E = 0$) being larger for the smooth glass. There are no indications for individual phonon signatures which would show themselves in peak position shifts for different TOF recording angles. This observation, combined with the fact that the distribution is centred around the elastic peak, supports our diffuse elastic scattering model, though a diffuse inelastic scattering component in the spectrum cannot be excluded.

Supplementary Note 3.2: Diamond measurements

Supplementary Fig. 2a shows rocking curve measurements for two different diamond samples, namely micro and intermediate diamond. There are no signs of diffraction or resonance scattering over the whole range. Below in Supplementary Fig. 2b the Michelson contrast from HAS is plotted together with the Michelson contrast via SHeM. Again, there is very good agreement between the two.

Supplementary Fig. 2c shows the energy-resolved TOF spectra for a room temperature beam for a range of incident scattering conditions. The most probable energy in the case of Knudsen flux scattering is indicated as a blue line in all plots. As can be seen, there is again no indication of Knudsen flux. Similarly to the glass samples, the only clear feature for diamond is a slight change in the overall intensity with the elastic scattering signal being larger for the microdiamond, due to large smooth patches that are also clearly visible in the SheM and AFM.

Supplementary Note 3.3: Au on Si measurements

HAS measurements were recorded for the 9 ± 2 Å , 26 ± 6 Å, and 235 ± 58 Å Au on Si samples, as well as for the pure Si surface. Supplementary Fig. 3a and 3b show the rocking curve and corresponding SHeM contrast measurements, respectively. Supplementary Fig 3c shows the energy-resolved TOF spectra. Despite the differences in materials, the exact same trends can be observed as for the previously measured systems.

Comparing the TOF spectra for in and off-specular conditions, no recognisable TOF peak position shift for either of the two Au on Si layer thicknesses can be found. Likewise, there is no peak position shift visible for pure Si, which indicates that there is no visible individual phonon signature in any of the TOF spectra. As above, there is no indication of helium atoms undergoing a thermal equalisation with the sample surface.

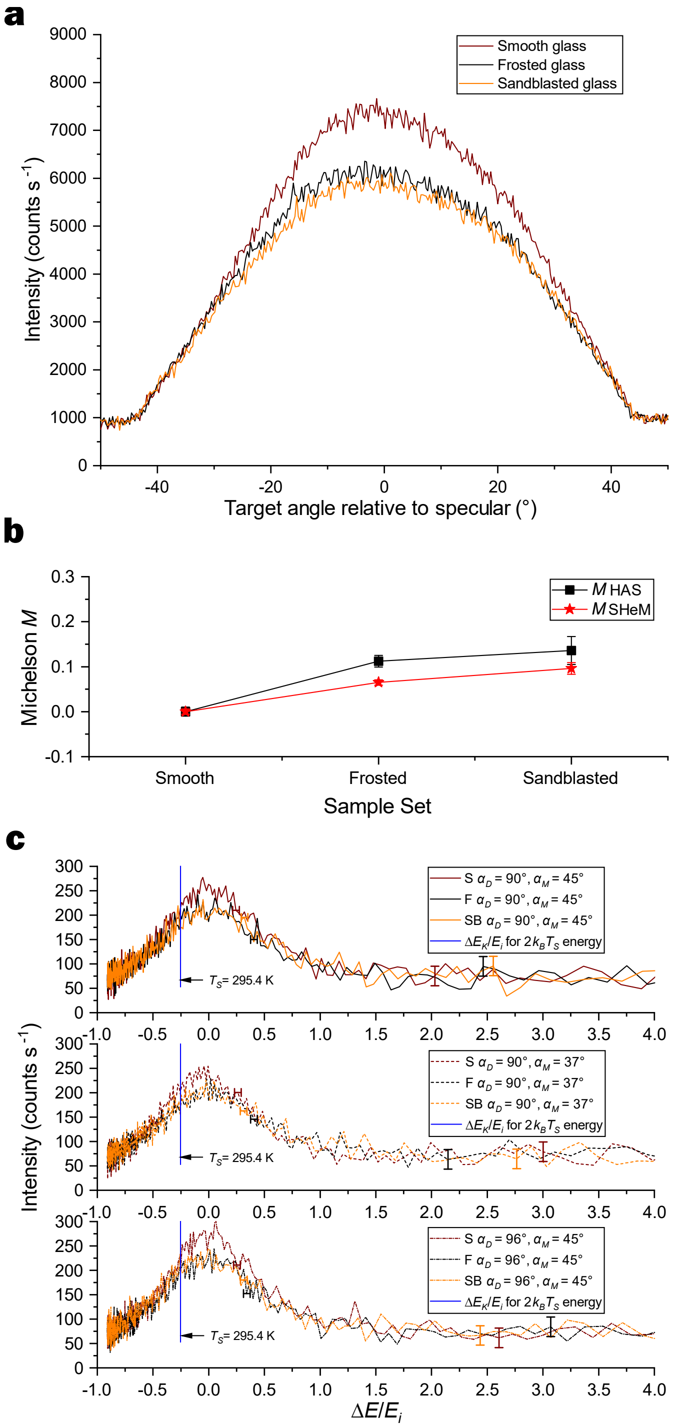

Supplementary Fig. 1 | HAS experimental data for the glass sample series. a. Calibrated and background corrected HAS rocking curves for the smooth ('S', dark red), frosted ('F', black) and sandblasted ('SB', orange) glass samples. The reflected helium intensity around 0° was highest for the smooth sample and lowest for the sandblasted surface. These data correspond well to the trend in the recorded intensities obtained via the SHeM (namely the smooth, frosted and sandblasted samples in order of most to least bright in the SHeM scans - see Fig. 2 and Supplementary Fig. 4a). Note that all measurements are background corrected by recording a measurement with a flag blocking the beam prior to the detector chamber. **b.** Comparison of the Michelson contrast values obtained from HAS (black rectangles) and SHeM (red stars) experiments. HAS error bars were calculated through error propagation from the measurement uncertainty in the HAS rocking curves. **c.** Energy-resolved TOF spectra for the glass sample series. Top graph: specular condition, with $\alpha_M = \alpha_D/2$; middle graph: sample manipulator rotated to off-specular with $\alpha_D = 37^{\circ}$ and $\alpha_M = 90^{\circ}$; and bottom graph: detector rotated to off-specular with $\alpha_D = 96^{\circ}$ and $\alpha_M = 45^{\circ}$. The most probable energy for Knudsen Flux scattering is indicated as a blue line in each graph. Note that the elastic signal ($\Delta E = 0$) is strongest for the smooth glass. Horizontal error bars in **c.** are calculated via error propagation of the uncertainty influenced measurement values for the flight time t_f and the sample temperature T_S. $E_i = 68.25 \pm 0.25$ meV (with error $t_f = \pm 2\mu s$) and $Ts = 294.5 \pm 1K$. Other horizontal errors were found to be $\Delta E_k = \pm 0.035$ meV, $\Delta E_k / E_i = \pm 0.01$ meV, $\Delta E = \pm 2$ meV (with $t_f = \pm 15\mu s$) and $\Delta E/E_i = \pm 0.03 meV$. The vertical uncertainly was evaluated from the maximum deviation of the TOF signal to the peak height of a Gaussian fitted through the measured TOF curve. The vertical error bars represent the maximum deviation for all 9 presented curves (±20 s^{-1}). For a clear representation of the TOF curves the original TOF signals were smoothed by adjacent averaging with a 10 points window.

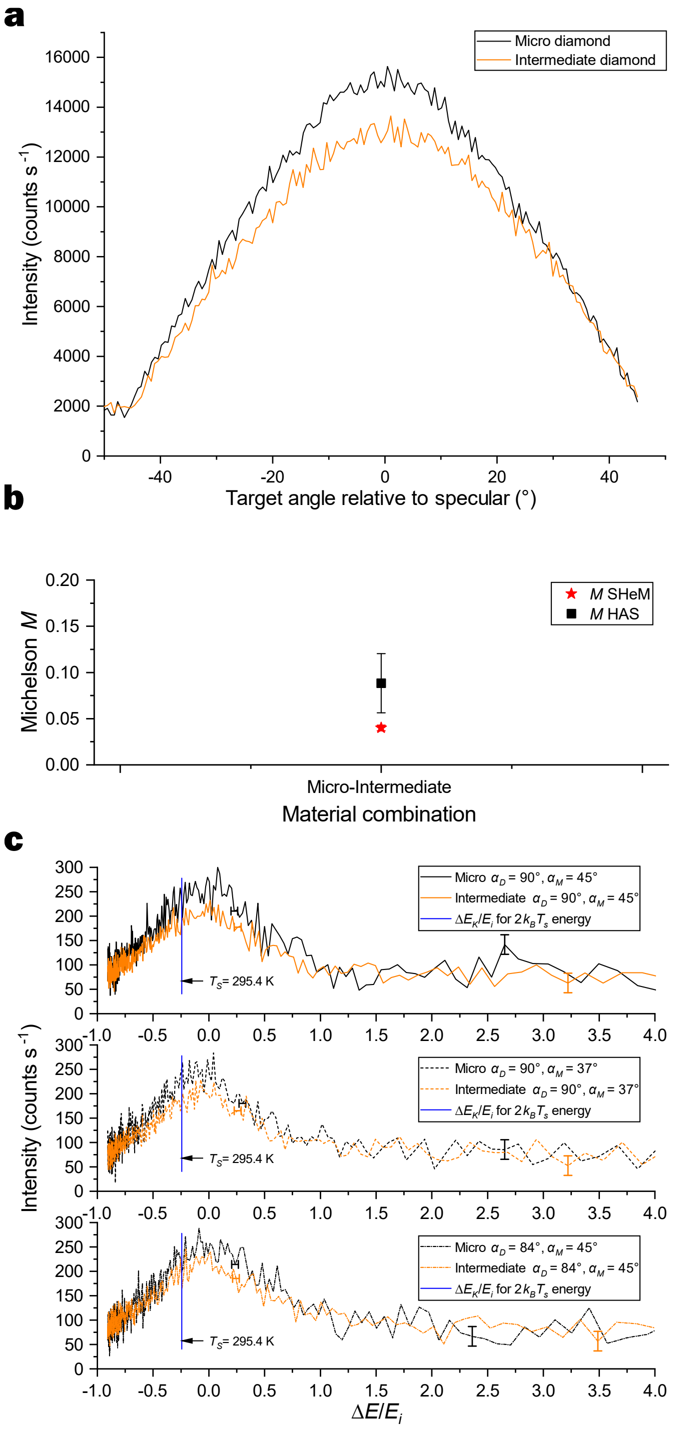

Supplementary Fig. 2 | HAS experimental data for the diamond sample series. a. Calibrated and background corrected HAS rocking curves for the micro (black) and intermediate (orange) diamond samples. The reflected helium intensity around 0° was higher for the micro than for the intermediate diamond. This trend corresponds well to the recorded SHeM intensities for the different roughness diamond surfaces, with the micro diamond showing up brighter than the intermediate diamond (see Fig. 2a and Supplementary Fig. 4b). Note that all measurements are background corrected by recording a measurement with a flag blocking the beam prior to the detector chamber. **b.** Comparison of the Michelson contrast values obtained from HAS (black rectangles) and SHeM (red stars) experiments. HAS error bars were calculated through error propagation from the measurement uncertainty in the HAS rocking curves. **c.** Energy-resolved TOF spectra for the diamond sample series. Top graph: specular condition, with $\alpha_M = \alpha_D/2$; middle graph: sample manipulator rotated to off-specular with $\alpha_D = 37^\circ$ and $\alpha_M = 90^\circ$; and bottom graph: detector rotated to off-specular with $\alpha_D = 96^\circ$ and $\alpha_M = 45^\circ$. The most probable energy for Knudsen Flux scattering is indicated as a blue line in each graph. Note that the elastic signal ($\Delta E = 0$) is strongest for the micro diamond sample. Horizontal error bars in c. were calculated through error propagation of the uncertainty influenced measurement values for the flight time t_f and the sample temperature T_S. $E_i = 67.20 \pm 0.25$ meV (with error $t_f = \pm 2\mu s$) and $T_s = 294.5 \pm 1K$. Other horizontal errors were found to be $\Delta E_k = \pm 0.035$ meV, $\Delta E_k/E_i = \pm 0.01$ meV, $\Delta E = \pm 2$ meV (with $t_f = \pm 15\mu s$) and $\Delta E/E_i = \pm 0.03$meV. The vertical uncertainly was evaluated from the maximum deviation of the TOF signal to the peak height of a Gaussian fitted through the measured TOF curve. The vertical error bars represent the maximum deviation for all 9 presented curves (±20 s^{-1}). For a clear representation of the TOF curves the original TOF signals were smoothed by adjacent averaging with a 10 points window.

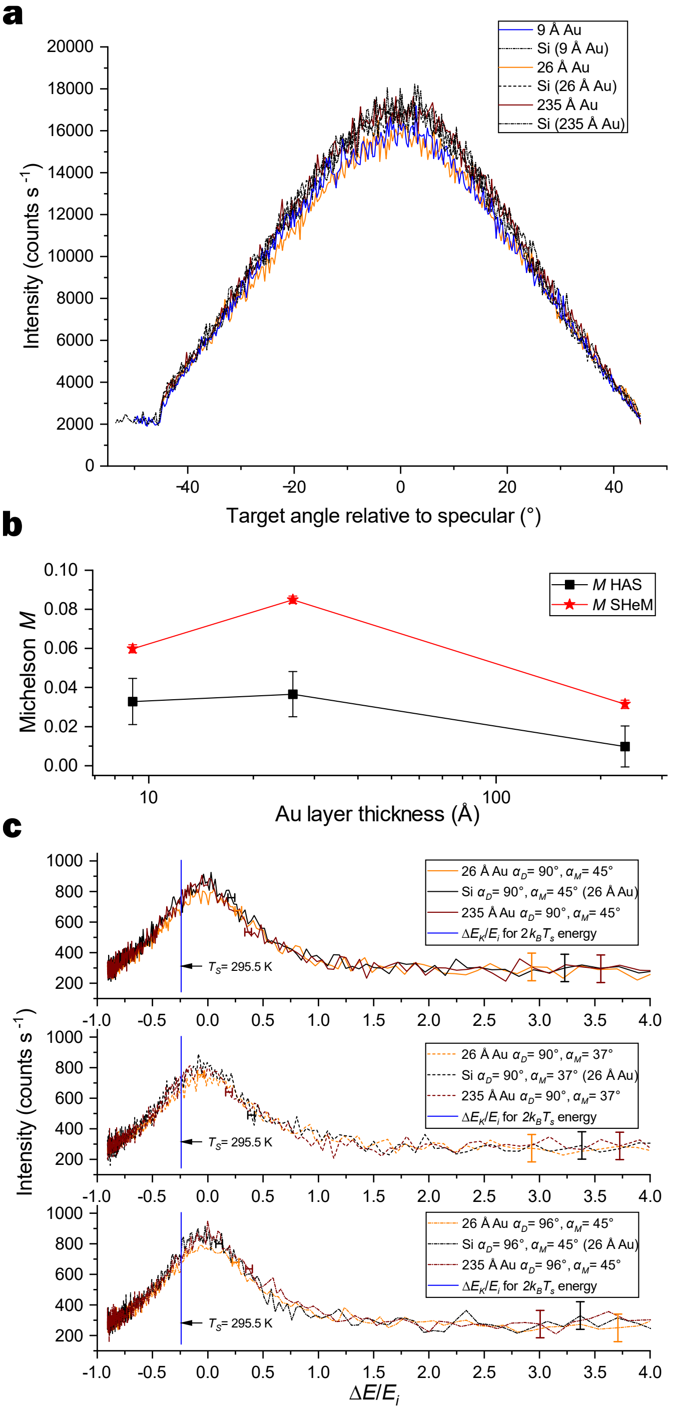

Supplementary Fig. 3 | HAS experimental data for the Au on Si sample series. a. Calibrated and background corrected HAS rocking curves for the 9 Å (blue), 26 Å (orange), and 235 Å (dark red) Au on Si samples, as well as their respective pure Si surfaces (black). The reflected helium intensity around 0° was the same for the 235 Å and the pure silicon surfaces, in good agreement with the (low) contrast observed in the SHeM. There is a clear reflected intensity loss between the pure silicon and the 9 Å gold layer, which corresponds well to the stronger contrast measured with the SHeM for this sample. The biggest intensity difference was found for the 26 Å gold layer as compared to the silicon reference surfaces; corresponding to the peak in the SHeM contrast curve (see Fig 3b). Note that all measurements are background corrected by recording a measurement with a flag blocking the beam prior to the detector chamber. **b.** Comparison of the Michelson contrast values obtained from HAS (black rectangles) and SHeM (red stars) experiments. HAS error bars were calculated as for Supplementary Fig.2 and 3. **c.** Energy-resolved TOF spectra for the Au on Si sample series. Top graph: specular condition, with $\alpha_M = \alpha_D/2$; middle graph: sample manipulator rotated to off-specular with $\alpha_D = 37^\circ$ and $\alpha_M = 90^\circ$; and bottom graph: detector rotated to off-specular with $\alpha_D = 96^\circ$ and $\alpha_M = 45^\circ$. The most probable energy for Knudsen Flux scattering is indicated as a blue line in each graph. Horizontal error bars in c. were calculated as for Supplementary Fig. 2 and 3. $E_i = 67.20 \pm 0.25$ meV (with error $t_f = \pm 2 \mu s$) and $T_s = 294.5 \pm 1K$. Other horizontal errors were found to be $\Delta E_k = \pm 0.035$ meV, $\Delta E_k/E_i = \pm 0.01$ meV, $\Delta E = \pm 2$ meV (with $t_f = \pm 15 \mu s$) and $\Delta E/E_i = \pm 0.03$meV. The vertical uncertainly was evaluated from the maximum deviation of the TOF signal to the peak height of a Gaussian fitted through the measured TOF curve. The vertical error bars represent the maximum deviation for all 9 presented curves (±90 s^{-1}). For a clear representation of the TOF curves the original TOF signals were smoothed by adjacent averaging with a 10 points window.

Supplementary Note 4: SHeM beam temperature studies

As a separate way to test for inelastic scattering contributions to the recorded SHeM contrast, a helium probe temperature (energy) study was recorded for all investigated sample systems. Inelastic scattering is suspected to yield a change of Michelson contrast with a changing helium probe temperature.

Supplementary Note 4.1: Smooth & frosted glass

Supplementary Fig. 4a shows the recorded SHeM scans (inset images) and the corresponding evaluated Michelson contrast values M for the temperature study performed on frosted and smooth glass. No significant (outside the measurement error bars) change of the Michelson contrast was found.

Supplementary Note 4.2: Micro & intermediate diamond

When looking at the different diamond SHeM micrographs recorded with different helium beam temperatures as shown in Supplementary Fig. 4b, likewise no clear change in Michelson contrast can be observed. Again these results are expected, since the chemical composition of the micro and intermediate diamond is the same.

Supplementary Note 4.3: Thin 26 Å Au layer on Si

Supplementary Fig. 4c presents a SHeM temperature study recorded on the bottom left corner of the 3x3 mm masked 26 Å Au on Si sample. The evaluated Michelson contrast M for different helium beam temperatures shows no significant change of contrast as a function of temperature, much like that observed for the glass and diamond samples.

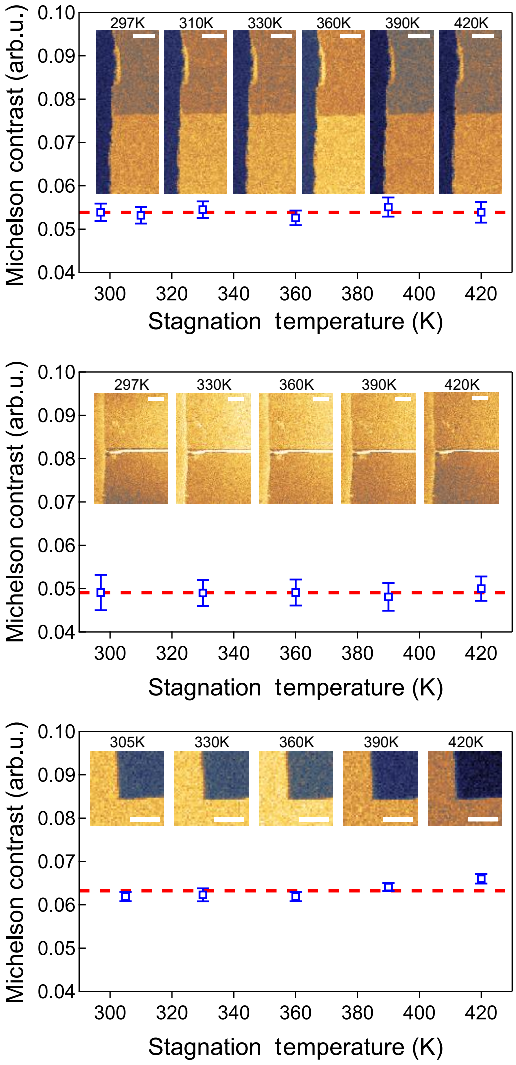

Supplementary Fig. 4 | SHeM temperature studies. Michelson contrast as a function of helium beam stagnation temperature for samples from the glass (**a.**), diamond (**b.**), and Au on Si (**c.**) series. Insets in each instance show the SHeM micrographs used to obtain the Michelson contrast values, with the micrographs in each experiment set utilising the same intensity range to allow for direct comparison. Red dashed lines indicate the mean Michelson contrast for each experiment set. Errors in the Michelson contrast are estimated from the standard deviation of the micrograph intensity via standard error propagation. **a.** SHeM temperature study for the Frosted (top of inset micrographs) and Smooth (bottom of inset micrographs) glass samples. No meaningful changes in Michelson contrast are observed as a function of beam stagnation temperature, as expected for samples with the same chemical composition. **b.** SHeM temperature study for the micro (top of inset micrographs) and intermediate (bottom of inset micrographs) diamond samples. Once more, no meaningful changes in Michelson contrast are observed as a function of beam stagnation temperature. **c.** SHeM temperature study measurements for the 26 Å Au on Si sample used in the experiments summarised in Fig. 3. While there is potentially a very minor evolution in the Michelson contrast at higher stagnation temperatures (namely, the final data point), given the measurement error it seems more likely that the sample exhibits no clear indication of inelastic scattering, despite the two different materials under investigation.

Supplementary Note 5: Discussion and conclusion

In Supplementary Fig. 5, a range of the presented TOF spectra have been normalised to show the same maximum intensity. We see that the shape of all TOF spectra are essentially identical, despite the different materials examined: gold, glass, silicon and diamond with very different atomic mass units. It is known that for a clean and ordered surface, the energy loss depends strongly on the mass ratio between the incident helium beam and the surface atoms and given the large differences (for example) in Au and Si mass, one would expect different shapes and peak positions. Supplementary Fig. 5 thus presents a very interesting result that highlights the sensitivity of HAS and SHeM. The helium atom interaction with the substrate is strictly surface sensitive. The reason that the TOF signal looks identical for all materials is with a very strong likelihood that the samples have been investigated without any cleaning. This means that we are seeing a multi-phonon background (diffuse inelastic scattering) from adsorbates on the samples (water, CO etc.). Because all samples are covered with the same adsorbates, the multi-phonon background looks the same for all materials. However, there is still a difference in the signal intensity close to specular between the samples, due to the diffuse elastic scattering, which reflects the topography of the individual samples and which we use in our model. This explanation is supported by the temperature studies presented in Supplementary Fig. 4, which show that the contrast does not change when the helium beam energy is changed.

Most materials will get covered by adsorbates over time so the trend described here should be more or less universal, except for a few types of materials such as graphene, which do not get easily contaminated by adsorbates. For such materials it will be necessary to describe the contrast with another model. Future work should include doing measurements on samples at elevated substrate temperatures where no or little adsorption is expected even over the prolonged measurement time required to obtain a SHeM image. With the present setup it was not possible to carry out such measurements.

We conclude that our experiments suggest that the diffuse elastic scattering is mainly responsible for the contrast in the SHeM images presented and thus our model is well suited to describe the images. We note that the rougher the surface the better the agreement will be between the Lambertian scattering model and our diffuse elastic scattering model.

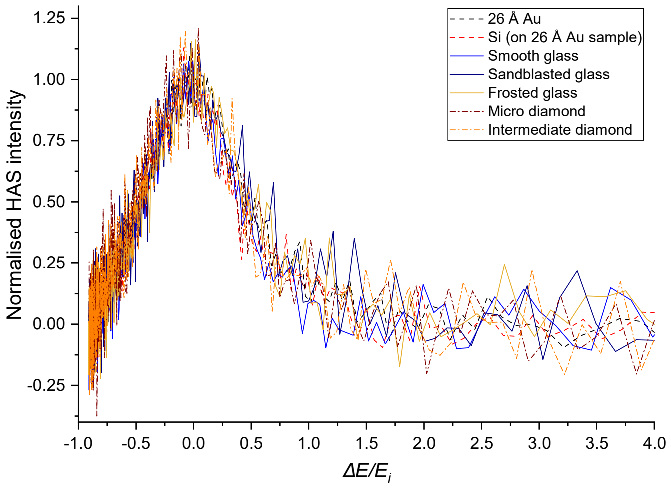

Supplementary Fig. 5 | Energy-resolved TOF spectra for various investigated samples. Normalised off-specular TOF spectra for all investigated materials, measured with $\alpha_D = 90^{\circ}$ and $\alpha_M = 37^{\circ}$. There is no meaningful difference observed between the recorded spectra for all investigated materials.

Supplementary Discussion 2: AFM analysis

Supplementary Note 6: Hurst parameter

The Hurst parameter [12] was calculated from the AFM maps via a custom MATLAB script modified from earlier work found in the literature [13]. The calculation first involves extracting a 1D profile by drawing an arbitrary line segment across the AFM map, with h_r the recorded height at position r along said line. We then define z_r as

$$z_r = \log_{10} h_r \quad . \tag{3}$$

To compare the 1D profile with an offset version of itself, we define an arbitrary lag in position along the line profile R. The variance of z_r as a function of R is given by

$$Var(R) = \langle |z_{r+R} - z_r|^2 \rangle \quad , \tag{4}$$

and thus, the Hurst parameter H is defined by

$$Var(R) \sim R^{2H} \quad . \tag{5}$$

When h_r can be described by geometric Brownian motion – that is, their correlation lengths tend to zero – $Var(R)$ will vary linearly as a function of R, and so $H = 0.5$. Hurst parameters greater than 0.5 indicate long range order (such as large surface facets) and values less than 0.5 tend to show the presence of mean-reverting structures (such as a jagged profile that has a consistent mean plane).

The MATLAB script works to avoid any bias in the calculation of the Hurst parameter by taking many line segments (and hence 1D profiles) through the AFM map. A schematic representation of the calculation process is shown in Supplementary Fig. 6 with the script calculating H along a series of lines emanating from each corner of the map. $Var(R)$ is then determined along each of these line segments, with the final recorded H calculated from the mean of the individual Hurst parameters.

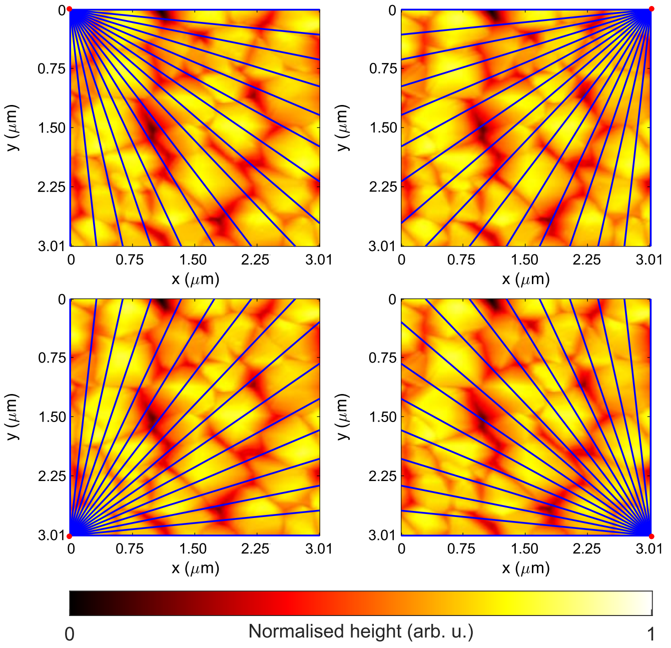

Supplementary Fig. 6 | A schematic of the process used to calculate the *H* parameter for an AFM height map. The origin of the line segments (shown in blue) is shifted to each corner of the map, in order to remove potential bias from an arbitrary choice of 1D profile. The final recorded *H* parameter is extracted from the mean of the individual *H* parameters calculated from each of the line segments. Note that only 10% of the line profiles used in the actual calculation are shown here for reasons of clarity.

Supplementary Note 7: Correlation length

The correlation length was calculated from the AFM maps via a custom MATLAB script. First, the 2D autocorrelation function (ACF) is computed using the standard MATLAB *xcorr2* library [14]. Subsequently, the 1D, single-tailed radial ACF was determined by taking line segments through the 2D ACF (see Supplementary Fig. 7a for a schematic representation). The correlation length proper was then calculated by taking the mean of the distance at which the individual 1D ACFs (Supplementary Fig. 7b) dropped to $1/e$ of their initial value. The uncertainty in the final correlation length's value was calculated as twice the standard deviation of all individual ACF correlation lengths (the 95% confidence interval).

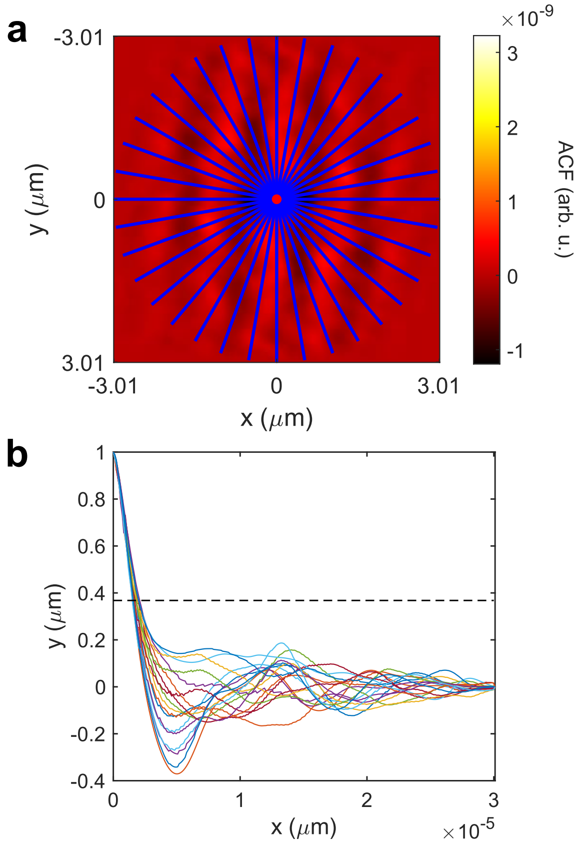

Supplementary Fig. 7 | A schematic representation of the process used to calculate the correlation length for an AFM map. a. the 2D autocorrelation function of the relevant sample is generated before a series of line segments (blue) are populated about the origin and used to determine 1D radial autocorrelation functions. **b.** plots of the derived 1D radial autocorrelation functions. The dashed line indicates the value of 1/e used to define the correlation length.

Supplementary Note 8: AFM analysis of material systems

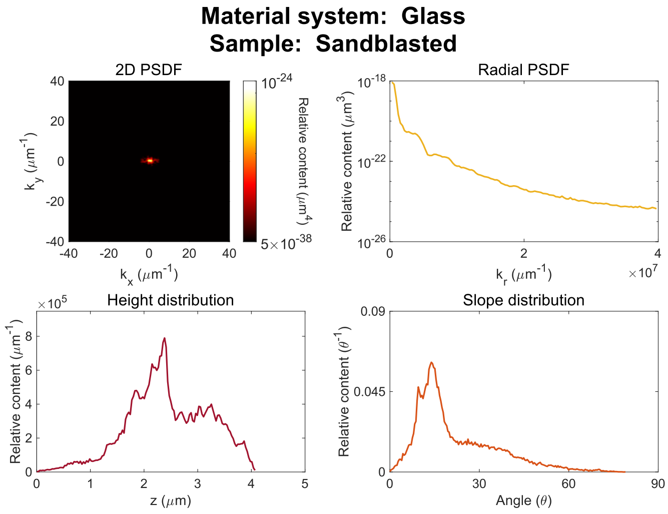

Supplementary Fig. 8 | AFM analysis for the sandblasted glass system. The 2D power spectral density, the 1D radial power spectral density, the distribution of height and the distribution of slopes, as calculated using the Gwyddion software package.

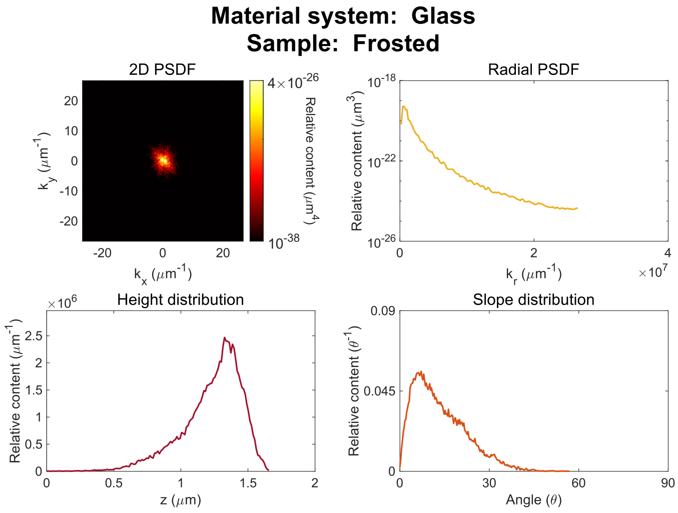

Supplementary Fig. 9 | AFM analysis for the frosted glass system. The 2D power spectral density, the 1D radial power spectral density, the distribution of height and the distribution of slopes, as calculated using the Gwyddion software package.

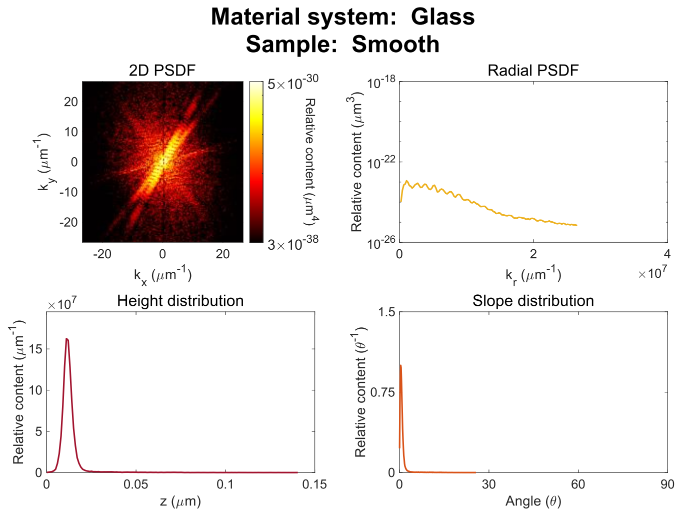

Supplementary Fig. 10 | AFM analysis for the smooth glass system. The 2D power spectral density, the 1D radial power spectral density, the distribution of height and the distribution of slopes, as calculated using the Gwyddion software package.

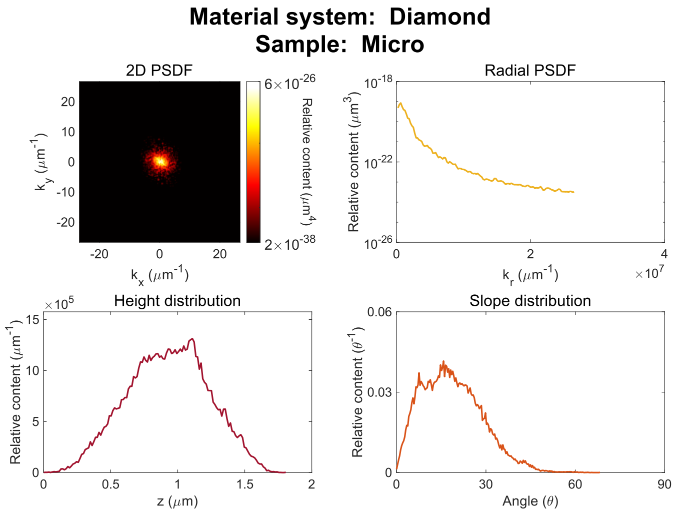

Supplementary Fig. 11 | AFM analysis for the micro diamond system. The 2D power spectral density, the 1D radial power spectral density, the distribution of height and the distribution of slopes, as calculated using the Gwyddion software package.

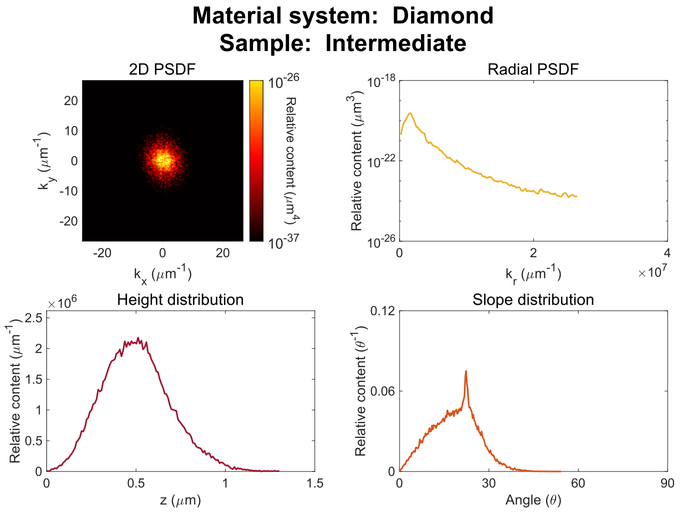

Supplementary Fig. 12 | AFM analysis for the intermediate diamond system. The 2D power spectral density, the 1D radial power spectral density, the distribution of height and the distribution of slopes, as calculated using the Gwyddion software package.

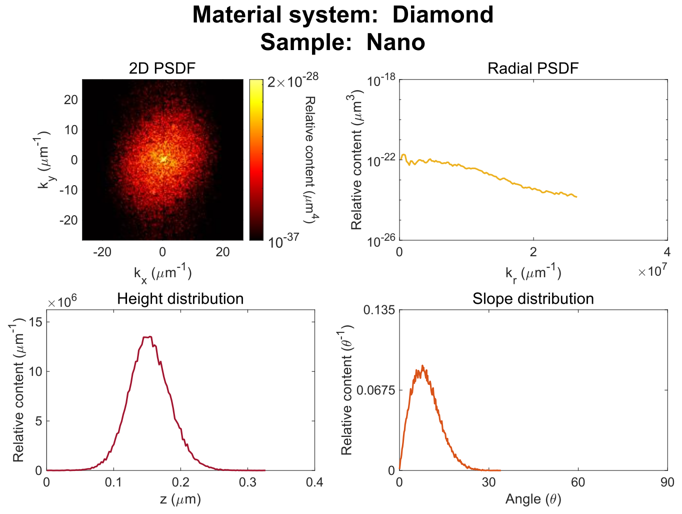

Supplementary Fig. 13 | AFM analysis for the nano diamond system. The 2D power spectral density, the 1D radial power spectral density, the distribution of height and the distribution of slopes, as calculated using the Gwyddion software package.

Supplementary Discussion 3: Basic reconstruction

Standard AFM calibration gratings (TipsNano TGZ1, TGZ2, TGZ3 and TGZ4) were purchased and imaged via AFM and with the SHeM. Similarly to the methodology used in Fig. 3 (thermally-evaporated gold on silicon), three of the samples (TGZ1, TGZ2, and TGZ4) were used alongside the facet-scattering model to generate a calibration curve for the SHeM intensity for this material system. Based on this calibration curve, the experimental SHeM intensity of the remaining sample (TGZ3) was used to predict the depth of this "unknown" grating. Supplementary Fig. 14 shows the calibration curve along with its 95% confidence interval. Reconstructing the unknown datapoint, the micrograph intensity predicts a grating depth of 566 ± 37 nm, well-matched to that obtained from the certified depth (528 ± 5 nm) (PTB, National Metrology Institute).

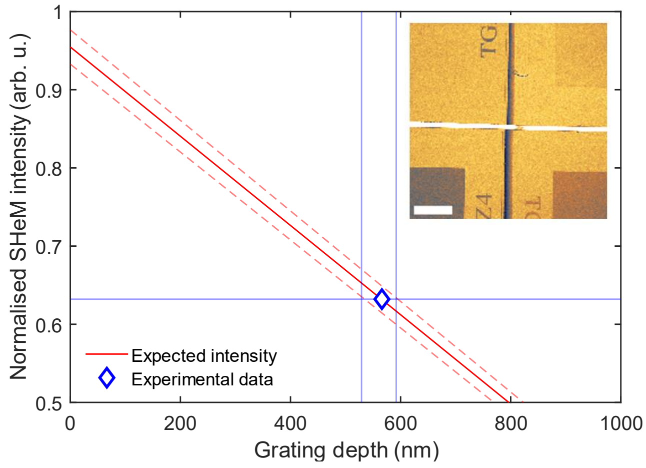

Supplementary Fig. 14 | Normalised SHeM intensity curve used to reconstruct the feature height of an unknown grating. The red line represents the expected intensity for a given grating depth, based on the facet-scattering model normalised using the known gratings (TGZ1, TGZ2, and TGZ4), with the dashed red lines representing the 95% confidence interval. The blue diamond shows the experimentally-recorded normalised SHeM intensity for the unknown grating (TGZ3), with the measurement error estimated from the standard deviation in the collected signal. Note that resultant error bars are smaller than the blue diamond marker. The vertical lines highlight the range of reconstructed grating depths (566 ± 37 nm). Inset shows the SHeM micrograph used to obtain the experimental Michelson contrast values for the gratings; TGZ1, TGZ2, TGZ3 and TGZ4 in clockwise order from top left corner of the micrograph. Micrograph scale bar 1mm in length.

Supplementary References:

1. Holst, B. et. al. Material properties particularly suited to be measured with helium scattering: selected examples from 2D materials, van der Waals heterostructures, glassy materials, catalytic substrates, topological insulators and superconducting radio frequency materials. *Phys. Chem. Chem. Phys*. **23**, 7653 (2021).
2. Benedek, G. and Toennies, J.P. *Atomic Scale Dynamics at Surfaces: Theory and Experimental Studies with Helium Atom Scattering*. Springer Series in Surface Science, 63 (Springer Berlin / Heidelberg, 2018).
3. Holst, B. and Bracco, G. *Probing Surfaces with Thermal He Atoms: Scattering and Microscopy with a Soft Touch.* Springer Series in Surface Sciences vol. 51, 333–365 (Springer, Berlin, Heidelberg, 2013).
4. Palau, A.S., Bracco, G. & Holst, B. Theoretical model of the helium pinhole microscope. *Phys. Rev. A* **94**, 063624 (2016)
5. Palau, A. S., Bracco, G. & Holst, B. Theoretical model of the helium zone plate microscope. *Phys. Rev. A* **95**, 13611 (2017).
6. Lambrick, S.M., Bergin, M., Jardine, A.P. & Ward, D.J. A ray tracing method for predicting contrast in neutral atom beam imaging. *Micron* **113**, 61-68 (2018).
7. Greenwood, J. The correct and incorrect generation of a cosine distribution of scattered particles for Monte-Carlo modelling of vacuum systems. *Vacuum* **67**, 217–222 (2002).
8. Bergin, M., et al. Observation of diffraction contrast in scanning helium microscopy. *Sci. Rep.* **10**, 2053 (2020).
9. O'Keefe, D. R. & Palmer, R. L. Atomic and molecular beam scattering from macroscopically rough surfaces. *J. Vac. Sci. Technol.* **8**, 27 (2001).
10. Morse, M. D. *Atomic, Molecular, and optical Physics, Atoms and Molecules*. in (ed. F.B. Dunning, R. G. H.) vol. 29B 21–47 (Academic Press, Inc., 1996).
11. Knudsen, M. *The Kinetic Theory of Gases. Some Modern Aspects.* (Methuen's Monographs on Physical Subjects) (London, Methuen; New York, Wiley, 1950).
12. Camargo, A. L. P. et al. Estimation of statistical properties of rough surface profiles from the Hurst exponent of speckle patterns. *Appl. Opt.* **59**, 5957-5966 (2020).
13. Aste, T. (2022). Generalized Hurst exponent (https://www.mathworks.com/matlabcentral/fileexchange/30076-generalized-hurst-exponent), MATLAB Central File Exchange. Retrieved June 14, 2022.
14. https://au.mathworks.com/help/signal/ref/xcorr2.html
